# Management of multi-surface ocular burns caused by molten iron

**DOI:** 10.1016/j.tcr.2023.100925

**Published:** 2023-09-02

**Authors:** Ekta Singla, Ujjwal Prakash Jha, Shruti Muralidharan, Rohan Bir Singh, Parul Ichhpujani

**Affiliations:** a Department of Ophthalmology, Government Medical College and Hospital, Chandigarh, India; b Massachusetts Eye and Ear, Department of Ophthalmology, Harvard Medical School, Boston, USA; c Department of Ophthalmology, Leiden University Medical Center, 2333ZA Leiden, the Netherlands

**Keywords:** Eye trauma, Burns, Ocular surface burns

## Abstract

Ocular thermal burns are medical emergencies that require immediate intervention before the standard management protocol, which involves obtaining a detailed history and performing an ophthalmic examination. In this case report, we report the clinical manifestations of ocular burns caused by molten iron and the steps taken for good clinical outcomes. The patient presented with an inferior epithelial defect and limbal and lower lid ischemia at four hours post-injury. Over the course of treatment, due to non-resolving epithelial defect and increased superior lid notching, amniotic membrane transplantation (AMT) and lid repair by pentagon wedge excision were performed. Following AMT, the corneal surface completely healed with residual opacity and neovascularization. Additionally, limbal ischemia was significantly reduced with the restoration of normal lid anatomy. Corneal burns initiate a cascade of inflammatory reactions disrupting the balance between pro- and anti-angiogenic factors, leading to corneal neovascularization. The eyelid damage can lead to necrosis of tissues with eschar formation and eventually quantitative tissue loss. Therefore, timely intervention is the key to the successful management of ocular burns.

## Introduction

Ocular thermal burns are medical emergencies requiring immediate treatment before initiating the standard protocol. Thermal burns are typically associated with exposure to boiling water, firework explosions, steam, or molten metal [1]. Early presentation in patients with ocular thermal burns can vary from small corneal epithelial defects to corneal melts, loss of conjunctival epithelium, and limbal ischemia. The late complications include symblepharon formation, secondary glaucoma due to inflammation of trabecular meshwork, corneal perforation, and limbal stem cell deficiency (LSCD). The primary aim of the interventions is to suppress inflammation, promote healing, and prevent corneal tissue melting [2].

## Case report

**Fig. 1:**
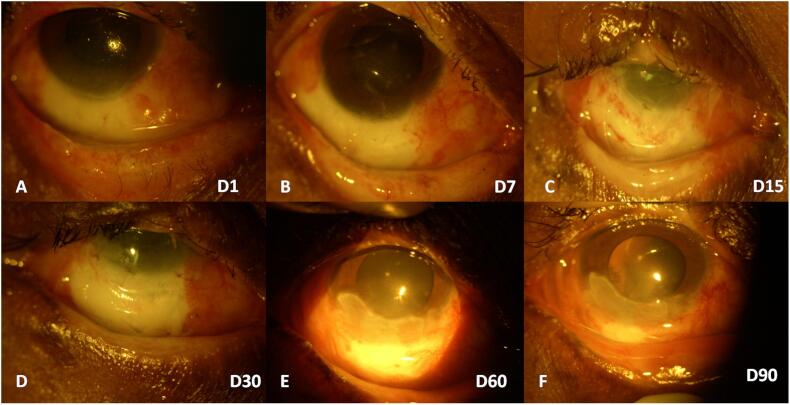
(A–F): Serial photographs from the day of injury to 3 months post-injury.

**Fig. 2:**
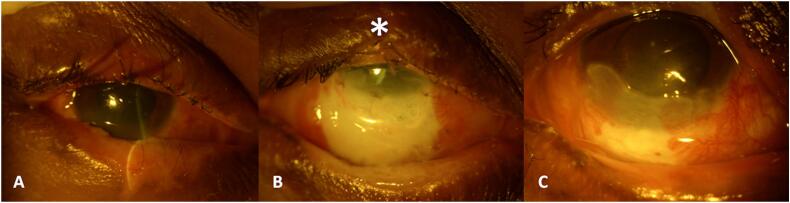
(A) Lid margin hyperemia with singeing of eyelashes, (B) ischemia of the lower lid margin, (C) healed lid margin.

The case report describes the successful management of unilateral ocular molten iron burn, which was medically and surgically treated using an amniotic membrane graft (AMG) and lid reconstruction. A factory worker in his third decade presented to the ophthalmology emergency room with pain, hyperemia, and reduced vision in his right eye, 4 h after accidental injury with hot molten iron (Fig. 1A). The patient had received copious irrigation with Balanced Salt Solution (BSS), and some particulate foreign bodies were removed at the primary healthcare center before being referred to our center. At presentation, his unaided visual acuity was counting fingers at 1 m in the right eye (OD) and 20/20 in the left eye (OS). The slit lamp examination of OD revealed lid edema with ischemia of lower lid margins (Fig. 2A), diffuse bulbar conjunctival congestion, corneal stromal edema, and an epithelial defect in the inferior half of the cornea and conjunctiva. The limbal ischemia involving 5 clock hours from 4 to 9 clock hours was also noted (Fig. 1A). The digital intraocular tension assessment was normal. The fundus could not be visualized and the B-scan ocular ultrasonography was non -contributory. We diagnosed the patient with grade 3 ocular surface burn (as per Dua's classification) in the right eye. The patient's OS anterior and posterior segment examinations were normal.

## Medical and surgical management

The patient was counseled regarding the guarded visual prognosis and need for hospital admission, followed by surgical intervention using an AMG. As the patient did not provide consent for the same, he was prescribed topical betamethasone 0.1 % hourly, moxifloxacin 0.5 % four times daily, atropine three times daily, preservative-free carboxy-methyl cellulose 0.5 % hourly, and hypromellose 2 % eye gel along with oral ascorbate (Vitamin C) 500 mg and Doxycycline 100 mg twice daily. On subsequent visits, the patient had symptomatic improvement. However, after one week of therapy, conjunctival and corneal epithelial defects increased in size (Fig. 1B), along with pronounced lower lid ischemia and telangiectasia with upper lid notching (Fig. 2B). The patient was informed about guarded visual prognosis and surgical intervention in the form of onlay (freshly harvested, COVID negative) AMG (Fig. 1C) with interrupted 8–0 vicryl (polyglactin 910) sutures under local anesthesia were performed. AMG was sutured deep into the fornix inferiorly to prevent symblepharon formation, followed by a bandage contact lens placement.

The lid margin changes (ischemia, excoriation, and notching) were markedly significant in the upper eyelid compared to the lower lid. Therefore, an upper lid repair using Tenzel semi-circular advancement flap with lateral canthotomy with cantholysis to reduce tension was performed, followed by direct closure using interrupted silk 5–0 suture under local anesthesia. On subsequent follow-up, lid margins were well apposed with AMG and Bandage Contact lens in place. The lid sutures were removed ten days post-lid reconstruction. The patient was followed up at weekly intervals (Fig. 1D).

## Outcomes and follow up

At the two-month follow-up, corneal neovascularization was observed, and the patient was prescribed Cyclosporine 0.1 % eye drops twice daily (Fig. 1E). At the last (three-month post-injury) follow-up, the visual acuity improved to 20/200, and the intraocular pressure was 15 mmHg (using handheld Tonopen). The cornea healed with residual opacity and vascularisation from 4 to 8 clock hours. The limbal ischemia was reduced to only 2 clock hours (Fig. 1F). The inferior lid margin was healthy (Fig. 2C). The patient was suggested simple limbal epithelial transplantation (SLET) but refused intervention in his only ‘normal’ seeing eye (OS).

## Discussion

Ocular burns are a commonly reported cause of ocular emergencies, accounting for approximately 8–18 % of ocular trauma. These burns are primarily accidental, occurring at work or home, and are seen more commonly in young males compared to females [3]. Thermal burns rarely cause severe ocular complications because of protective mechanisms such as blink reflex, Bell's phenomenon, and reflex protective movements of the head and arms. Effective and timely intervention can minimize permanent vision loss from thermal trauma. The involvement of the eyelids and lid margins is the most common ophthalmic injury. Eyelid damage can lead to necrosis of tissues with eschar formation and eventually quantitative tissue loss [4].

The severity of tissue destruction in ocular thermal burn depends primarily on four factors, i.e., the temperature of the agent, heat-retaining capacity of the material, area over which the heat is applied, and duration of contact [5]. The patient was exposed to hot molten iron with a high heat-retaining capacity and temperature ranging from 200°–1200 °C [6]. The direct contact occurred approximately 30 min before thorough irrigation. The cases reported in the literature are primarily associated with molten metal or glass-like agents with high melting temperatures (1000 °C) and significant heat-retaining capacity to cause severe burns and tissue opacification due to deeper corneal penetration [7,8].

In the past, ocular burns were classified using Roper Hall classification [9,10]. Dua and colleagues proposed a new classification with six grades incorporating conjunctival and corneal components to determine the extent of limbal involvement [1]. The patient had five clock hours of limbal ischemia with nearly 30 % bulbar conjunctival involvement and was categorized as Grade III per Dua's classification of ocular and surface burn. In such cases, timely intervention is the key to successfully managing the ocular burn. The initial aim is to copiously irrigate the area to remove particulate matter. Ringer's lactate and BSS are more effective than normal saline as these solutions have similar osmolarity to aqueous humor and thus prevent corneal swelling [3]. The goal of the subsequent interventions is to promote surface epithelialization and healing, reduce inflammation, prevent superadded infection, and maintain normal intraocular pressure. Preservative-free lubricants promote re-epithelisation, topical steroids help control inflammation, and topical antibiotics are indicated for antimicrobial prophylaxis. The trauma initiates a cascade of inflammatory responses disrupting the balance between pro- and anti-angiogenic factors, eventually leading to corneal neovascularization. Cyclosporine inhibits T-cell proliferation and thus plays a critical role in preventing the same [3]. Therefore, after initial suppression of the cytokine storm associated with trauma using oral corticosteroids, the patient was prescribed cyclosporine eye drops. Additionally, oral or topical ascorbate (Vitamin C) promotes epithelial healing and helps prevent stromal necrosis. A combination of surgical intervention with medical therapy may be performed in cases involving extensive damage and necrosis. AMG helps in promoting epithelialization while decreasing the inflammation around the limbal area [11]. Tamhane and colleagues compared conservative therapy alone versus conservative treatment with AMG and noted that the latter helped reduce pain and promote early re-epithelialization [12]. In this case, the patient was advised surgical intervention considering the extent and severity of the thermal injury, but it was initially managed medically due to refusal to consent. After a week of fluctuating responses to medical therapy and non-resolving epithelial defect, an AMG was done.

Jackson's burn wound model divides burns into three zones based on pathophysiology [4]. The central zone, i.e., the zone of maximum heat transfer, is known as the “zone of coagulation,” which is surrounded by the “zone of stasis” (i.e., zone of intense inflammation). It is a potentially salvageable area that can aggravate to full-thickness injury by infection, ischemia, or wound drying. The outermost “zone of hyperemia” (inflammation) is the site of minimal injury and undergoes early spontaneous recovery. Hence, burns initially presenting as dermal/partial thickness may progress to full-thickness injury if appropriate and timely measures are not taken [4]. Although the evidence in the literature suggests a waiting period of at least three weeks from injury to undertake a surgical lid repair, some evidence also outlines the benefit of early lid repair (within seven days of injury) to reduce corneal exposure and superadded purulent keratitis [13,14]. In this case, repair using a Tenzel semicircular advancement flap was performed at two weeks post-injury due to non-resolving lid ischemia and preventing further progression of the lid notching.

## Patient consent

Written consent to publish the case report was obtained from the patient.

## Funding

No funding or grant support.

## ICJME

All authors attest that they meet the current ICMJE criteria for authorship.

## Declaration of competing interest

None.
